# Synthetic biology: Learning the way toward high-precision biological design

**DOI:** 10.1371/journal.pbio.3002116

**Published:** 2023-04-26

**Authors:** Shohei Kitano, Ciai Lin, Jee Loon Foo, Matthew Wook Chang

**Affiliations:** 1 NUS Synthetic Biology for Clinical and Technological Innovation (SynCTI), National University of Singapore, Singapore; 2 Synthetic Biology Translational Research Programme, Yong Loo Lin School of Medicine, National University of Singapore, Singapore; 3 Department of Biochemistry, Yong Loo Lin School of Medicine, National University of Singapore, Singapore

## Abstract

Since its inception, synthetic biology has overcome many technical barriers but is at a crossroads for high-precision biological design. Devising ways to fully utilize big biological data may be the key to achieving greater heights in synthetic biology.

## Abstract 2

The field of synthetic biology has come a long way in the past 20 years but is at a crossroads for high-precision biological design. This Perspective looks at how machine learning could help synthetic biology to achieve greater heights.

This article is part of the *PLOS Biology* 20th Anniversary Collection.

Though the term “synthetic biology” was coined over a century ago in 1912, the field has only relatively recently matured in the last two decades. Today, synthetic biology is summed up as an approach that aims to rationally reprogram organisms with desired functionalities through engineering principles. Taking inspiration from the assembly of electronic circuits, the discipline aspires to alter biological behaviors with genetic circuits constructed using standardized biological parts. Indeed, initial efforts have shown the feasibility of reprogramming cellular behaviors for novel functionalities. Early successes such as a genetic toggle switch [1], an oscillator [2] and a cell-cell communication circuit [3] teased the possibility of someday creating programmable organisms that can change their behaviors and function autonomously depending on environmental stimuli. Following these breakthroughs, synthetic biology’s progress has accelerated in the past decade—giving rise to applications in various areas, from therapeutics to biomanufacturing. For instance, microorganisms that sense and kill cancer cells have been developed [4], as well as cell factories that autonomously optimize their metabolic pathways according to their conditions [5]. The remarkable rate of technological advancements has driven synthetic biology to grow more and more interdisciplinary in the past twenty years. Given these developments so far, synthetic biology promises to deliver future technologies that can resolve crucial problems currently faced by our society.

Synthetic biology employs the “design-build-test-learn” (DBTL) cycle as its development pipeline. In the past decade, the “design” and “build” stages have been propelled by massive improvements in DNA sequencing and synthesizing technologies, leading to significant reductions in cost and turnaround time. In 2007, sequencing a human genome required an estimated USD$10 million, falling to around USD$600 today. This cost-effectiveness has allowed us to sequence whole genomes of organisms and amass vast amounts of genomic information in databases that form the basis for re-designing biological systems. Taking advantage of the easing of DNA synthesis costs and the wealth of available genomic data, it is now possible to synthesize and harness genetic parts from organisms that we do not possess. Coupled with novel DNA assembly methodologies such as Gibson assembly [6], we have overcome the limitations of conventional cloning methods to enable the seamless assembly of combinatorial genetic parts, thus elevating our assembly capacity. Consequently, synthetic biologists can now even assemble entire chromosomes from chemically synthesized DNAs [6, 7]. Concurrently, the development of genetic toolkits and genome editing techniques has revolutionized synthetic biology, enabling the manipulation of a wide range of organisms, including non-model ones which were previously considered difficult to manipulate, and expanding the arsenal of organisms that can serve as chassis, or biological platforms, for synthetic biology.

Recent innovations in the “building” of biological systems have led to a drastic surge in the number of samples characterized in the “test” stage of the DBTL cycle. The increased rate of sample generation now exceeds the capacity of manual handling techniques, driving demand for high-throughput testing methods that use automation. As a result, biofoundries have been built worldwide, with several key facilities coming together to form the Global Biofoundry Alliance in 2019 [8]. In these biofoundries, a multitude of biological parts and systems can be built and tested rapidly through high-throughput automated assembly and screening methods. Such high-throughput technologies can then be leveraged by next-generation sequencing and mass spectrometry to collect large amounts of multi-omics data for cells at the single-cell level.

Despite overcoming technical barriers in the “building” and “testing” biological systems to generate enormous amounts of biological data, synthetic biologists have faced difficulties in learning from big biological data. So far, the DBTL cycle’s “learning” stage has proved challenging due to the complexity and heterogeneity of biological systems, the interactions between different components, as well as variations in experimental setups. Although synthetic biologists can sufficiently decipher data to create draft blueprints of the desired biological systems, many still resort to top-down approaches based on likelihoods and trial-and-error to determine the optimum design. This deviates from the aspiration of synthetic biology to rationally design organisms from characterized genetic parts. To bring the discipline to new heights, it is critical to have breakthroughs in processing and “learning” from big datasets.

One way to facilitate the “learning” stage of the DBTL cycle is by tapping into computational power for mathematical modelling to process and understand biological data. Modelling has enabled the simulation of not only simple systems but also complicated biological ones, such as the whole-cell metabolism of *Mycoplasma genitalium* [9]. However, while these simulations provide detailed and comprehensive insights for learning and re-designing biological systems, developing predictive biological models requires a profound knowledge of all essential reactions in such organisms. Yet, biological processes in cells are often highly dynamic and inscrutable “black boxes”. Hence, even modelling is unable to fully capitalize on the big data generated in synthetic biology to comprehend organisms, especially when applied to complex and heterogeneous environments such as the human gut and large-scale bioreactors.

Recently, a more advanced and powerful computational approach known as machine learning (ML) has gained traction in synthetic biology [10] for potentially promising to overcome the DBTL cycle’s “learning” bottleneck. ML processes big data and provides predictive models by choosing appropriate features to represent a phenomenon of interest and uncovering unseen patterns among them. Indeed, ML has already been used to improve biological components, such as promoters [11] and enzymes [12], at the genetic part level. This is relatively easy to achieve since there is a sufficient dataset size for ML. To advance synthetic biology further, ML needs to facilitate the system-level prediction of biological designs possessing desired characteristics by elucidating the associations between phenotypes and various combinations of genetic parts and genotypes. As explainable ML advances, we anticipate the provision of both predictions and reasons for the proposed design, deepening our understanding of biological relationships and accelerating the “learn” stage of the DBTL cycle (Fig 1). Thus, ML presents an attractive avenue for distilling complex biological information and brings us closer to synthetic biology’s aspiration to establish core design principles for the rational engineering of organisms. While ML’s potential to revolutionize synthetic biology should be further explored, it should be noted that the technique cannot predict everything from data. To lay the groundwork for the extensive application of ML in synthetic biology, common standards for designing and generating ML-friendly data should be established and collaborations cultivated among dry- and wet-laboratory researchers globally—building upon decades of concerted efforts from the synthetic biology community.

**Fig 1:**
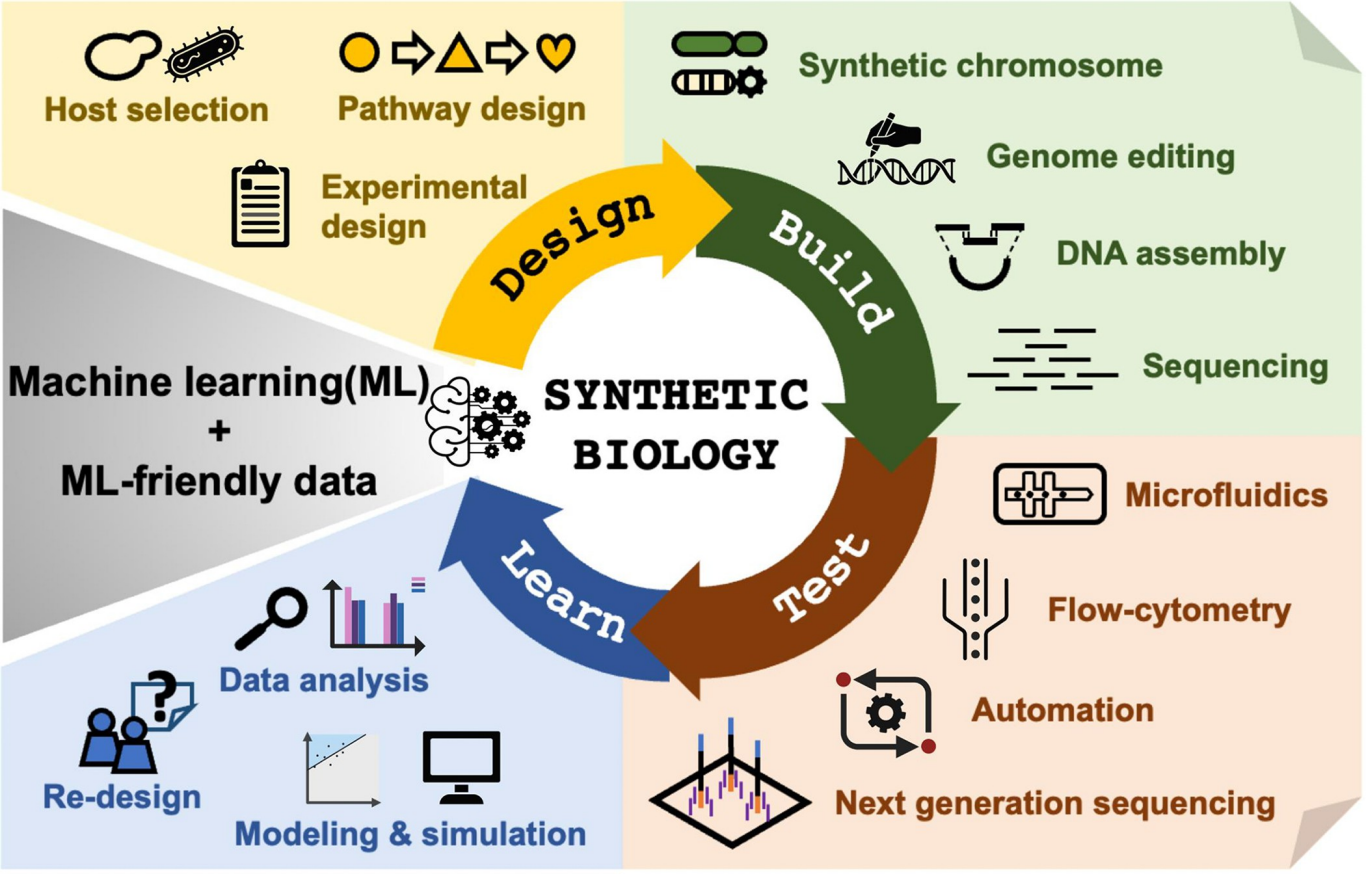
Schematic of a machine learning-driven “design-build-test-learning” (DBTL) cycle in synthetic biology. The DBTL cycle is a framework in synthetic biology for developing organisms with desired functionalities. Over the years, the bottlenecks associated with the technologies depicted in the figure have gradually been resolved, enabling the advancement of each stage in the cycle. However, developments in the “learn” stage continue to lag. Machine learning can bridge the gap between the “learn” and “design” stages to further accelerate the DBTL cycle. This figure was created using clipart from BioRender.com.

With ML-friendly data on hand and a deepened understanding of complex biological systems enabled by ML, we foresee that next-generation precision biological design could soon become a reality. As ML processes the big data we have amassed, our enhanced understanding of complicated biological systems will pave the way for precision synthetic biology and achieve a new paradigm of predictive cell biodesign. By integrating ML into the synthetic biology workflow, we can potentially generate precise metabolic blueprints for engineering robust organisms with predictable and defined autonomous behaviors that could then be applied in real-world settings, such as sustainable chemical production. For instance, we can engineer microbes that sense fermentation conditions in real-time to optimize their metabolic flux and modulate their stress response accordingly, accomplishing high productivity and robustness in an industrial setting. Through a build-to-learn approach, precision design can also advance synthetic genomics and aid in unraveling the mechanisms behind complex multifactorial genetic disorders caused by polygenic mutations. ML-driven understanding will then allow for the development of accurate models that can be used for clinical studies and precision therapies, such as diagnostic and therapeutic microbes that can identify diseases *in situ* and produce drugs *in vivo* based on the diagnoses. Ultimately, we envision that ML will play a key role in debottlenecking the DBTL cycle, finally allowing the full potential of synthetic biology to be unleashed.
